# Are mHealth Interventions to Improve Child Restraint System Installation of Value? A Mixed Methods Study of Parents

**DOI:** 10.3390/ijerph14101122

**Published:** 2017-09-26

**Authors:** Linda Fleisher, Danielle Erkoboni, Katherine Halkyard, Emily Sykes, Marisol S. Norris, Lorrie Walker, Flaura Winston

**Affiliations:** 1 The Children’s Hospital of Philadelphia, Philadelphia, PA 19104, USA; klhalkyard@gmail.com (K.H.); sykese@email.chop.edu (E.S.); WINSTON@email.chop.edu (F.W.); 2 National Clinician Scholars, University of Pennsylvania, Philadelphia, PA 19104, USA; erkoboni@mail.med.upenn.edu; 3 Creative Art Therapies and Counseling, Drexel University, Philadelphia, PA 19104, USA; marisol.s.norris@gmail.com; 4 Safekids Worldwide, Washington, DC 20037, USA; lwalker@safekids.org

**Keywords:** child restraint systems, child safety education, mobile health, web-based interventions, stakeholder engagement

## Abstract

Childhood death from vehicle crashes and the delivery of information about proper child restraint systems (CRS) use continues to be a critical public health issue. Safe Seat, a sequential, mixed-methods study identified gaps in parental knowledge about and perceived challenges in the use of appropriate CRS and insights into the preferences of various technological approaches to deliver CRS education. Focus groups (eight groups with 21 participants) and a quantitative national survey (N = 1251) using MTurk were conducted. Although there were differences in the age, racial/ethnic background, and educational level between the focus group participants and the national sample, there was a great deal of consistency in the need for more timely and personalized information about CRS. The majority of parents did not utilize car seat check professionals although they expressed interest in and lack of knowledge about how to access these resources. Although there was some interest in an app that would be personalized and able to push just-in-time content (e.g., new guidelines, location and times of car seat checks), content that has sporadic relevance (e.g., initial installation) seemed more appropriate for a website. Stakeholder input is critical to guide the development and delivery of acceptable and useful child safety education.

## 1. Introduction

Injuries to passengers in motor vehicle crashes remain a leading cause of death for children. The correct use of appropriate child restraint systems (CRS) in the rear seat reduces this injury risk [1], yet families continue to transport children in ways that do not optimize their protection despite extensive public awareness and educational campaigns and laws. The delivery of information about proper CRS use remains a critical public health issue. The topic of child passenger safety (CPS) is relevant to most parents, with information needs changing across time and context [2,3]. Appropriate motor vehicle restraint is a safety practice that changes with the child’s age [4], and misuse by parents remains high across all age groups [5,6,7]. While the Haddon Matrix tells us that the reasons for this misuse is likely multi-factorial [8], a well-cited body of literature has supported a significant knowledge and technical skill deficit by parents as a potential cause [9]. Research has shown that although parents and caregivers report having correctly installed a CRS, objective observations and assessments often indicate incorrect CRS installation [10,11].

As mobile technology becomes ubiquitous [12], the opportunity exists to deliver families broad-based yet adaptive and timely child passenger safety information to help address this gap in CRS practice. The use of evidence-based, theoretically grounded content delivered in convenient technical form is necessary but not sufficient to ensure success. Best practices in health communication, behavior theory and adaptive technology design should be applied to ensure that the messages and technology are relevant and positively impact caregiver behavior. Central to this approach is parent/caregiver engagement about the issue of protecting children in motor vehicles, the value of technology for prevention and the types of specific technological approaches and tools that are most useful.

Health communication studies have clearly demonstrated parental preference for tailored over generic messages [13]. This is in accordance with the elaboration likelihood model [10], which demonstrates that people are more likely to actively process and engage with personally relevant information [14,15,16]. Tailored communication provides information unique to prescribed characteristics of the parent, child or family. A 2016 qualitative study by Peng et al. found users wanting designs that were interactive, social and individualized [17]. This is in line with a movement toward applications that are not only tailored toward the users’ needs at a single time point, but also across time as the users’ needs change. Increasingly popular, these adaptive models match the user’s spatial and temporal needs with targeted content, eliminating the static nature of previous designs in mobile health applications [18,19,20]. This design allows content to be intervention-determined and context-triggered rather than user-determined and triggered [21,22,23]. While these tailored applications hold vast potential for promoting health behavior change [23], limited research has explored how adaptive mobile health applications can be used to reach parents, particularly related to injury prevention.

Therefore, the Safe Seat study aimed to match technological solutions to meeting the CPS needs of families/caregivers. Through the application of a sequential, mixed-methods approach, the study (reviewed by CHOP IRB and considered exempt) identified gaps in parental knowledge about and perceived challenges in the use of appropriate child restraints in motor vehicles while also gathering perceived value and preferences in technology for delivering CRS education. Using focus groups, data from a national sample of caregivers and adaptive technology development approaches, these findings provide guidance to program planners and researchers who are developing emerging and technology-based educational interventions to address childhood injury prevention. As a result, our study, “Safe Seat”, is a model to discuss parents’ perceptions of adaptive mobile health applications. This exploratory sequential mixed methods pilot [24] was designed to both provide direct insight on the development of an adaptive, native mobile health application for parents related to CPS and to inform development of future adaptive applications around other health topics. 

## 2. Materials and Methods

### 2.1. Focus Groups

#### 2.1.1. Aim

A series of focus groups were conducted with parents and caregivers of young children to assess perspectives on and use of existing tools to guide car seat installation, as well as to explore the acceptability of using a mobile app to guide car seat installation. 

#### 2.1.2. Eligibility and Recruitment

Parents/caregivers were recruited from five outpatient pediatric primary care practices connected to a large children’s hospital in the greater Philadelphia area. Primary care practices were chosen based on geographic location to ensure variability in participant race/ethnicity, community setting and socioeconomic status. A list of 400 potentially eligible participants was generated for each of the participating practices for review by study personnel. Criteria for inclusion in the initial sample pool included: parent/guardian has a child that is a patient at the participating practice, the child is between ages 0 and 5 (inclusive). Participant eligibility was determined according to the age of the child (child must be between ages 1 and 2, or 4 and 5, inclusive), child race, child medical insurance type (private or Medicaid), parent report of transporting a child in a car seat at least 2 times per week, parent report of installing any type of car seat at least 5 times in the last 6 months, and parent ability to travel to the local pediatric practice. Eligible parents/caregivers were invited to participate by mail and telephone. Parents and caregivers who contacted or were contacted by study staff and screened to be eligible were enrolled and scheduled for a focus group until an initial enrollment goal of at least 40 participants was achieved. The study was conducted in accordance with the Declaration of Helsinki, and the protocol was approved by the Ethics Committee of FWA0000459.

#### 2.1.3. Study Procedures

Upon arrival on the day of the focus group, participants completed a brief demographic questionnaire, which also gathered their child passenger restraint use patterns, as well as personal mobile device usage. Following the group session participants received a prepaid $20 gift card for their participation. Each of the eight focus groups were one hour in duration.

#### 2.1.4. Approach 

The focus group interviewers were developed by the research team (Linda Fleisher, Katherine Halkyard, Flaura Winston, and Marisol Morris), based on literature review and extensive research conducted at CHOP. The focus groups were led by the lead author who has training and extensive experience in focus group facilitation. The participants were recruited through each primary care practice and were not familiar with the research team. The focus groups were conducted at each of the participating clinics and therefore convenient for participants. A phenomenological research approach was employed for the conduct of the focus groups to explore participants’ experiences, perceptions, and concerns about CRS and their opinions on using technology to assist in car seat installation. Phenomenological research practices focus on the study of an identified phenomenon or experience from a group of individuals’ perspectives. Consumer knowledge and experiences installing car seats and utilizing digital and mobile educational resources for car seat installation, the phenomenon at hand, was explored with emphasis on parent/caregivers’ subjective experiences and personal interpretation of car-seat installation as well as their current and projected use of car seat installation resources, especially online and mobile resources.

Focus group recordings were transcribed and analyzed using inductive thematic analysis [25] to identify patterns across the dataset. Three reviewers conducted initial coding and then coding conferences were held to discuss and resolve discrepancies. In the first phase of analysis, apparent themes were identified from an initial review of the data. Focus group recordings were then transcribed and read in entirety by members of the research team, who identified initial codes. The transcripts were then imported to NVivo qualitative software (QSR International Pty Ltd., Melbourne, Australia) where data was further coded and themes and subthemes emergent across the dataset were identified and refined. Data were once again read in their entirety, along with accompanied memos, to ensure the themes identified were congruent with dataset and portrayed participants’ experience of the phenomenon. Lastly, themes were defined, categorized, and named.

### 2.2. National Survey

#### 2.2.1. Aim

Based on focus group data analysis, an online survey was developed to be administered to a national audience of parents and caregivers of young children using car seats. The aim of this survey was to obtain a broader, quantitative perspective on parents’ barriers and facilitators to installing a CRS correctly, resources utilized to guide CRS installation, and perspectives on using a mobile app to guide CRS installation.

#### 2.2.2. Eligibility and Recruitment 

Survey eligibility criteria included: participant is at least age 18 and older, English-speaking, parent/guardian/routine caretaker of child between 0 and 8 years old, and cares for at least one child that uses a car seat. Participants were compensated $1.00 for completing the survey. 

#### 2.2.3. Approach 

Survey topics were selected to expand on qualitative themes gathered from the focus group data, such as caregivers’ role in the installation process, parents’ assessment of perceived risks and benefits of installing a car seat correctly, parents’ attitudes about current resources available to help install a car seat, and parents’ values regarding content and features that could be included in a mobile app to guide CRS installation. Survey items addressing these topics were adapted from other validated CPS measures in the literature [26,27,28,29]. To establish content validity of the instrument, a panel of CPS and mobile app design experts from the Center for Injury Research and Prevention at Children’s Hospital of Philadelphia, Safe Kids Worldwide, and Drexel University Applied Informatics Groups (N = 5) reviewed and offered suggestions for revision. 

The final survey was constructed for online administration using Qualtrics^TM^ (a commercial survey software) (Qualtrics, Seattle, WA, USA; Provo, UT, USA) and was administered online through Amazon.com’s crowdsourcing labor site, Mechanical Turk (MTurk) (www.mturk.com). MTurk is a service to “crowdsource” labor intensive, yet simple tasks that can be performed on a computer. Literature review of studies using MTurk as a recruitment tool showed that MTurk users are generally younger adults (18 to 30 years old) who are single or have small families, mostly female, and of lower income compared to traditional study samples [30]. A preliminary review of the literature indicates that the majority of studies using MTurk are related to product marketing [31] and that this study is among the first to utilize Mechanical Turk to recruit parents of young children.

A preliminary version of the survey was launched on Mechanical Turk to obtain initial population sample distribution, as well as to obtain feedback on survey readability and flow from MTurk users. The survey was revised based on initial results and comments. A final 52-item survey was developed to assess parents’ utilization of and general experiences with installing a child’s car seat, as well as perspectives on using a mobile app as a resource to obtain information about CRS installation and CRS safety. Descriptive statistics were run for all survey responses.

## 3. Results

### 3.1. Focus Groups

#### 3.1.1. Sample Demographics

Fifty-seven parents or caregivers were initially enrolled and scheduled via telephone, with 21 parents or caregivers attending and participating in the eight in-person focus groups (37% participation rate). Groups had two to five participants each. The majority of participants were young parents or legal guardians (62% between 18 to 34 years old), female (86%), and African American (48%), with at least some college education (62%) (Table 1). 

The focus group survey indicated that most parents/guardians reported having only one child using a car seat at that time (66.6%), specific car seat brand and model (71.4%). Frequency of car seat installation in the last six months varied greatly, with most parents reporting that they installed their car seat 2–4 times (33.3%) or more than 11 times (28.6%). Focus group discussions later clarified that the frequency of car seat installation was highly dependent on both the type of seat being used (i.e., infant seats have semi-permanent bases that remain in the vehicle) and how often the car seat must be moved from one vehicle to another.

All participants owned a mobile phone (100%), with the majority owning a smartphone (90.5%). Similarly, most also owned a mobile tablet device (n = 16), with the majority using their mobile phones (85.7%) or a tablet (9.5%) to connect to the Internet most often. Mobile device ownership did not appear to vary by recruitment site. 

#### 3.1.2. Qualitative Themes

Key themes emerged about car seat installation and the value of technology (specifically, mobile phone apps versus Web). The complexity of car seat installation and the recognition of the role of parents/caregivers in this process were raised in the focus groups. These themes included the actors (first-time parents, skilled parents, expert parent, good enough parent or secondary caretakers), the child development timeline (prenatal, newborn, and infancy (rear facing); transition; and toddler (forward facing)), child car seat installation continuum (preparation, execution, evaluation and resolution), installation facilitators (community agents and resources, and technological resources), and installation barriers (lack of information, adjusting car seat). See Table A1 in Appendix A for a comprehensive table of these findings. In addition, the focus group discussion focused on the use of technology to assist in this process, and key themes included proponents of app use (easy accessibility, predictability), deterrents of app use (lack of technology savvy, occasional use, perceptions of value of web-based), and app content preferences (facts about car seat installation, vehicle compatibility, video tutorials). Participants discussed some of the resources available for installation, such as the manufacturers’ website and videos/tutorials online. One parent stated, “I did look at the ones that came from the manufacturer and then I looked at what regular people posted on YouTube”. Parents described the challenges with understanding the directions in the car seat manuals. In terms of using an app to facilitate care seat installation, there were varying viewpoints. Some indicated that an app would be good since “you are outside with your phone, not your computer” and there might be other caretakers, such as grandparents, that might need to install the seat. However, some caretakers do not use apps and would not download an app for something that they would only use occasionally. The focus group findings confirmed that car seat installation is a complex set of behaviors and requires considerable knowledge and skill. Moreover, although technology can facilitate increased knowledge and skill, there are differing views of what types of technology (app or web) would be most suitable. 

In addition to the general discussion, participants were asked a series of questions regarding opinions and preferences of using a mobile app to guide the car seat installation process. These questions focused on audience, ideal timing, value of using an app, features and deployment. Table 2 summarizes the findings from these discussions.

Participants were divided among proponents and opponents of using an app to guide car seat installation. Further discussion delineated the various advantages and disadvantages to using an app for this purpose. For example, proponents of app use thought an app would be easy to use, accessible and provide visual assistance. One participant said:

“When somebody else is going to need to install a car seat, for whatever reason… if it were my in-laws watching my children for some reason, or if you were my best friend or something I would direct them to something like that.”

Other participants suggested that an app would be helpful in real time:

“…You’re outside, so I have my phone with me. I wouldn’t have my computer outside with me. It would just be right there and I could check it and would be good to go…”

Another others pointed out that they like apps and know what to expect. 

However, other points of view were shared as well. Some participants stated that they did not use apps:

“I actually don’t use apps. I have a smartphone but I don't use apps …”

One of the key findings from these focus group discussions is that an app might not be ideal since car seat installation is very sporadic. As one participant stated:

“If you have an app on your cell phone… it’s something you do regularly but generally you don't install car seats regularly. You could search on the computer and look for some stuff and figure it out and then you are done.”

Another participant concurred:

“I think that like in the beginning it would be used but then how often would it actually be used because once you know how to install a car seat you know? Whether you're going from one car to another to another I mean it’s all the same and so that… I think it would be good for first timers you are learning how to use your car seat but after that how much would it really be used. Would the app cover all different types of car seat instillation cause that would be important, too?”

There was some agreement that a website might be better, but if the app had more features and more content, an app might also have appeal. These features include: FAQs or tips about car seat installation, as well as car seat safety rules, laws, and guidelines; car seat-vehicle compatibility; tutorial of installing various seats in various types of cars, as well how to adjust straps and position child in seat; and access to online forums or parent created car seat reviews. 

### 3.2. National Survey

Surveys were collected from a total of 2217 participants. Of this total, 750 participants were excluded who did not meet the survey eligibility criteria (parent of a child 0–8 years, greater than 18 years of age and English speaking). An additional subset of eligible participants was excluded from the final data set if they did not complete the full survey (n = 145), or did not meet attention check review (n = 71). Attention check review compared the responses to two ranking questions, with systematic responses to both ranking questions triggering further review of other rank order responses. Surveys with systematic responses (e.g., “123456789”, “213456789”, and “123456798”) to all rank questions were eliminated from analysis. Final analysis included 1251 participants.

*Sample Demographic Characteristics (Table 1).* A majority of participants was female (55.9%), with gender distribution for this sample more balanced than much of the Mechanical Turk literature [32,33]. The majority of participants were young parents or legal guardians (with 58.2% between 23 and 34 years old), Caucasian (81.3%) and with at least some college education (84.8%). There was a largely equal representation of participants from across the four U.S. regions (Northeast, South, Midwest, and West Coast), with about one quarter of participants representing each of these areas. 

#### 3.2.1. Key Findings

Parent responses to child seat use questions are summarized in Table A2 in Appendix A. The majority of participants saw themselves (70.2%) or the child’s co-parent (18.6%) as the primary installer of the car seat with over one-third of respondents re-installing a seat at least once per week. While a significant minority of participants reported challenges with installing a car seat (36.2% agree/strongly agree that “it is a hassle”), the majority reported that they always have their child ride in a seat, both in their own vehicle (95.8%) and in other vehicles (91.4%). 

The majority of participants used the manual that came with the car seat to learn how to initially it (54.6%), with experienced family members being the next most common source of information (18.8%). Many participants reported not having someone else check to see if the car seat was installed correctly, such as a certified car seat technician or trained safety professional. Only 30.4% of participants reported always having someone check their car seat installation. 

While most participants felt physically capable of proper car seat installation, barriers to installation appeared to be highly situational. Participants were asked to rank several circumstances in which it is most difficult to install a car seat; the highest ranked scenarios were when there is little space in the car, when there are many passengers in the car, if the child resists getting in the car seat, or when the individual is in a hurry. Even in these situations, the majority of parents/caretakers use the car seat for their child, with 91.6% of respondents indicating that they are always able to get their child buckled in the car seat even if he/she resists.

#### 3.2.2. Current Car Seat Installation Resources

Parent responses to current resources are summarized in Table A3 in Appendix A. While most participants indicated that it is easy to find information about car seat installation (82.2%), opinions varied on the satisfaction level of the information available (only 60.7% very satisfied). When asked how they look for information about car seats, the majority of participants reported using the Internet (73.5%) and most said that while on the Internet they use a search engine, such as Google, or use YouTube to look at videos. Manufacturer and non-profit websites such as SafeKids.org were the next most visited online resources.

#### 3.2.3. Using a Mobile App for Car Seat Installation

Parent digital use and perceptions are summarized in Table A4 in Appendix A. Similar to the focus group responses, almost all survey respondents owned a cell phone (96.7%), and, of those, most owned smartphones (90.5%). While 63.6% use their computers to access the Internet most of the time, 27.7% use their mobile phone, representing a shift from the focus group participants, who said they use their mobile phone most of the time to access the Internet. 

While 62.1% of respondents stated they had looked for health information online in the past, and 80.1% had downloaded an app onto their phone in the past, only 67.9% stated they would download an app about their child’s health and safety, and only 36.2% said they would definitely use an app specifically about car seats. Most participants agreed that a mobile app about car seats would be more convenient (55.8%) than the car seat manual, while 48.1% felt it would be simpler. However, while almost half said an app would be useful (46.9%), many also said an app would not be useful after the first few uses (48.8%). Over half of participants also said they would prefer to use a website rather than an app to get information about car seats (54%). 

Finally, participants were asked to indicate the types of features and content they would prefer to have in a hypothetical mobile app about car seats (Table 3). They were asked to rank information and features based on value and preference. In addition, they were asked to select and/or describe additional features and content they would prefer to have. Facts about the type of seat needed for the child, how to troubleshoot car seat installation, laws and policies about car seats, and how to buy the best car seat were top ranked information to include in the app, while FAQs and tips from experts were additional content that participants valued.

### 3.3. Triangulation of Sequential Qualitative and Quantitative Finding

Several emergent themes from the focus group data were used to inform the design of the national survey, including how parents used available resources for seat installation, ease with and frequency of seat installation and lack of follow-up on whether seats were installed correctly. The focus groups provided important insights about the perceived value of various technological approaches to support parents in car seat installation. Although there was a need for more information and support and mobile/web-based tools were of interest, it was not a consensus view. Quantitative MTurk national survey data allowed for these themes to be explored in further depth with attitudes, beliefs and practices in this larger population echoing those in the smaller qualitative population. Although there were differences in the age, racial/ethnic background, and educational level between the focus group participants and the national sample, there was a great deal of consistency in the need for more timely and personalized information about CRS. Largely, participants in both groups felt comfortable installing child safety seats for their children but turned to the user manual for initial information. Participants saw this information as single use and would often not return to the manual or other data sources even when installing the CRS in a new way or in a new vehicle. The majority of parents in both samples did not utilize professionals to check seat installation although they expressed interest in and lack of knowledge about how to access these resources. The use of apps vs. web-based resources raised important considerations regarding what type of information was needed to be in real-time as well as features, such as finding a car seat technician that would be enabled using mobile technology.

## 4. Limitations

There are a number of limitations to this study. Although the focus groups were conducted in both urban and suburban pediatric practices, they do not represent a national sample, and generalizability of these findings are limited. The national survey, conducted through MTurk, provided a broader representation of parents and caregivers. However, this was a convenience sample of parents and caregivers who have subscribed to participate in MTurk sponsored studies.

## 5. Discussion

The sequential mixed methods design of this study allowed for both qualitative exploration of these important issues to parents, as well as a deeper exploration of these themes in a larger population. While this study initially intended to inform the design of a mobile health educational tool for CRS use and installation, as a way to address the multi-factorial issue of child safety seat mis-use, qualitative and quantitative data revealed a different need. Parents raised concerns about a mobile application for this purpose alone, and would prefer to use a website to find information about CRS. Participants noted that information needed only once or twice for discrete periods of time, like car seat installation, was not best suited for a mobile app. Instead, mobile apps are better suited for more broad topics that may be accessed more frequently, such as to manage or look for information about their child’s health and safety. Parents noted that apps require significant and necessary space and data on their devices. For apps to be valuable, they must meet a repeated, unique need for the user related to the outcome of interest and derived from individual assessment. In our Safe Seat study, participants stated an app would be used infrequently. Although there was some interest in an app that would be personalized and able to push content (e.g., new guidelines, location and times of car seat checks), they saw content that has sporadic relevance (e.g., initial installation) as more relevant for a website, which could be accessed when needed. 

Results from this study were also summarized into a schematic diagram based on the phases of car seat installation, actors involved in the installation process, and changes in installation barriers/facilitators, as described in the focus group results. Features and content were mapped to this diagram based on their value rankings from the survey responses. The schematic diagram was used to produce a preliminary web app workflow design which was used to obtain feedback from target end-users and experts in the CRS safety field. As a result of these findings, it was decided that the final product of this formative research would not be a stand-alone mobile app, but rather a website, which could be easily accessed on either a mobile device or a computer. 

Following the conclusion of the Safe Seat study, Safe Kids Worldwide (www.safekids.org) developed a web-based app to assist parents who were overwhelmed by multiple decisions and choices when selecting a car seat for a child. The Ultimate Car Seat Guide (www.ultimatecarseatguide.com) from Safe Kids Worldwide (SKW), which is currently available and being widely disseminated, walks a parent through the four basic steps of buying, installing, fitting a harness and moving from one seat type to the next. Consumers have the option to create a personalized guided tour with more detailed information using their child’s weight and age. The Ultimate Car Seat Guide web app is designed to be used on a phone, tablet or computer. It is offered in English and in Spanish, and can be updated or amended relatively easily. The information offered in the Ultimate Car Seat Guide is informed by our deep experience working with families one-on-one for more than 20 years. Annually, SKW inspects approximately 120,000 car seats and in the past 20 years has inspected more than 2 million seats. SKW collects data on each seat inspected using certified child passenger safety technicians with a standardized checklist form that is later scanned and analyzed. SKW has more than 275 grass roots coalitions that work in the Buckle Up program. Overall, Safe Kids has solid data demonstrating parental misuse of car seats, errors in car seat selection and premature transitioning from one seat type to the next. While it would be ideal for every family to attend a car seat check with a certified child passenger safety technician, it is simply not possible. This online guide is created to make evidence-based, life-saving knowledge available to more families.

The Ultimate Car Seat Guide web app is now available in English and Spanish and the language in the Guide was recently evaluated by a plain reading specialist. It has been amended so both English and Spanish information is available to the person with low reading ability. SKW also plans to evaluate the impact of the Ultimate Car Seat Guide on caregiver knowledge. Questions addressing self-reported knowledge gain and demographics to allow a “scope of reach” assessment will be built into the Guide as a pop-up survey at the end of each section on the four basic steps. From September 2016 to end of July 2017, the site has received 76,690 visits with 53% from a mobile device, 40% from a desktop and 7% from a tablet device.

Central to the findings of the Safe Seat study was the use of our step-wise mixed methods approach, employing survey data gathered from Amazon.com’s crowdsourcing labor site Mechanical Turk (MTurk). While previous literature has shown MTurk’s implicit sample bias compared to other national or population based samples [34,35]. While our sample had greater females (55.9%), higher education (99.9% high school completion) and less diversity (80.1% Caucasian) than the latest census data, median income ($50,000) and parent age were in accordance with expected values. While these biases must be taken into account when interpreting the data, data on MTurk outcomes must be considered as well. Several studies have found the validity and reliability of these data to be equivalent, if not superior, to traditional methods [36,37,38]. Improved survey completion rates and validity of responses have been tied to factors such as compensation rate and survey length, which were taken into account in the design of this study.

While the findings from the study as they apply to child passenger safety must be taken into account in the design of future CPS interventions, it is important to note how the central themes from this work have broad applicability. First and foremost, the Safe Seat Study integrated stakeholder (parents, caregivers and injury prevention experts) perspectives throughout the process from the initial focus groups to the schematic technology diagram. Without this iterative process, our initial assumptions about the need for an app to improve car seat installation might have led to the development of an expensive tool that would not have been well utilized by parents and caregivers Formative research with stakeholder involvement is foundational to delivering effective and acceptable child safety educational interventions and facilitating a more sustainable and broader dissemination. Furthermore, within the public health community, we must look across specific content areas toward the broader themes emerging within literature on technology-based interventions. Both qualitative and quantitative data sources in the Safe Seat study addressed the fit of certain types of messages, such as those with a sporadic, recurrent need like child passenger restraint, with certain technology-based platforms. Consistent with previous studies, this serves as a call to action to pair formative research with strong evidence-based design pairing messaging type with technology platform. Stakeholder input is foundational to delivering effective and acceptable child safety educational interventions and facilitating a more sustainable and broader dissemination. 

## 6. Conclusions

In today’s increasingly digital age, a broad spectrum of platforms exists for digital health interventions, each of which is best suited to address different health topics. For example, a web app can be locally installed like a native app with the data pushed to the user, but it acts like a URL that the user can take with them. Examples include email clients, news readers, and task oriented applications. In this sense, a web app would also not store large amounts of data on the users’ device, an issue that was brought up for using apps in the survey responses but would be able to still use the innovative features of a smartphone, such as video playback, GPS, camera, and movement sensors. These types of platforms can be more adaptive addressing just-in-time issues, such as the changing ages of children, new guidelines, and car seat check locations. 

Furthermore, this research opens paths to explore how novel health communications methods can facilitate behavior change among diverse populations. Mobile technologies may better address gaps in language, literacy and cultural disparities since they are more interactive than print and can be easily adapted to different languages. This formative approach to technology-based intervention development can also be applied to other topics in child safety, such as vaccines, nutrition, and driving safety. 

## Figures and Tables

**Table 1:** Focus group participant demographic characteristics.

Characteristic	Focus Groups N = 21	National Survey N = 1251
Age (years)	N (%)	
18–34	13 (61.9%)	788 (63%)
35–54	5 (23.8%)	421 (33.7%)
55 and above	1 (4.8%)	154 (12.3%)
Missing	2 (9.5%)	0 (0%)
Gender	N (%)	
Male	3 (14.3%)	552 (44.1%)
Female	18 (85.7%)	699 (55.9%)
Race/Ethnicity	N (%)	
White	6 (28.6%)	1075 (81.3%)
Asian or Pacific Islander	1 (4.8%)	91 (6.9%)
Black or African American	10 (47.6%)	117 (8.9%)
Native American	0 (0%)	20 (1.5%)
Mixed Race	0 (0%)	37 (2.8%)
Other/Rather Not Say	2 (9.5%)	7 (0.5%)
Missing	2 (9.5%)	0 (0%)
Education Level	N (%)	
Less than High School	0 (0%)	1 (0.1%)
High School or Equivalent	5 (23.8%)	136 (10.9%)
Vocational School	0 (0%)	53 (4.2%)
Some College	8 (38.1%)	368 (29.4%)
College and beyond	6 (28.6%)	693 (55.4%)
Missing	0 (0%)	0 (0%)

**Table 2:** Caregiver preferences for features/content in mobile app.

Who Would Use the App?	When Would They Use the App?	How Useful Is an App?	What Should Be in the App?	Who or How Should It Be Advertised to Users?
Parents of children ages 0–6 First time moms Parents about to switch their child to a new seat Caregivers Family Babysitters Dads say they are less likely to use it but moms say dads would use it a lot	Before the baby is born When buying a new seat (process) or about to switch seats External caregiver installing First time installation with new seat When looking for car seat check points Troubleshooting In need of non-installation factoids/frequently asked questions (expiration dates)	Advantages On the go information Simple to use Accessible Provides direct info Easier to read Visual assistance Disadvantages Some preferred website Apps may be difficult to navigate Unfamiliarity with apps Not useful when installing—“keep checking your phone”	Car seat check locator and appointment scheduler What seat fit best in the car How to install in different types of cars Consumer product reviews Car seat laws and policies Types of car seats for height, weight, and age FAQ’s, safety myths vs. facts Expiration dates Traveling Online forum (social tools) Search function Multiple languages Spanish Videos and images Troubleshooting info and tools—inch and pinch test	Who: Not manufacturers Doctors in office CHOP Insurance companies Safekids Worldwide How: No brochures Parent magazines Website of CRS companies Poster in waiting room Prenatal and parenting classes

**Table 3:** Ranking of topics by national survey participants.

**Mobile App Content**	**N (% Ranked as #1)**
Switching my child from infant carrier/rear-facing seat to forward facing convertible car seat	128 (10.2%)
Switching my child from forward facing convertible seat to a booster seat	74 (5.9%)
Switching my child from a booster seat to a seat belt	44 (3.5%)
Facts about the type of car seat I need for my child based on their height, weight and age	333 (26.6%)
Laws and policies for car seats	200 (16%)
Where to find support to help me install my car seat	49 (3.9%)
How to talk to my child's doctor about car seats	20 (1.6%)
How to buy the best car seat for my child	197 (15.8%)
How to troubleshoot problems I have with installing the car seat in my car	206 (16.5%)
**Additional Features**	**N (% Ranked as #1)**
Frequently asked questions (FAQs) about car seats and car seat installation	983 (78.6%)
Top safety tips recommended by experts	913 (73%)
Myths and facts about car seats	737 (58.9%)
Facts about car seat expiration dates	720 (57.6%)
Using public transportation with a car seat	579 (46.3%)
Winter clothing and car seats	459 (36.7%)
Other	22 (1.8%)
Frequently asked questions (FAQs) about car seats and car seat installation	983 (78.6%)
Top safety tips recommended by experts	913 (73%)

## Appendix A

**Table A1:** Focus group themes and quotations.

Themes and Definitions	Category and Definitions	Corresponding Quotations
*Actors:* The key participants/stakeholders in the overall instillation process over the child-car seat development continuum (prenatal, infancy, toddlerhood, and the transition periods in between). Each points of the continuum are characterized by different attitudes, challenges, and behaviors.	*First-time parent (newbie/novice):* New to the instillation process and often feels anxious and overwhelmed by the responsibility and influx of information regarding car seat instillation. Lacks confidence in instillation ability, skill, and knowledge and may exhibit assistance seeking behaviors.	“…With the first baby you’re like so paranoid.” “It’s my first child so it’s like…I don’t know anything. So I'm like thank God for this print out cause I go over them every other day I think “this I’m not doing right, this ones…”
	*Skilled parent:* Has experience and demonstrates confidence in perceived installation knowledge/ability/skill and may exhibit assistance seeking behavior (to improve car seat instillation over time). Often this parent with 2 or more children has had practice and perceived mastery/confidence in the car seat instillation.	2nd child reference—“It was so much easier. You weren’t as worried. You were like Okay, they let me leave with a person last time… I didn’t kill them.” “… We had to buy a new seat for the older child because they are 22 months apart so we actually did that before we got the new child cause she was much bigger. We had that seat sitting around. We didn’t have to buy it. We knew. We knew we were going to do it again.”
	*Expert parent:* Has experience and demonstrates confidence in perceived installation knowledge/ability/skill and does not exhibit assistance seeking behaviors (to improve car seat instillation over time).	“I already had 3 boys, how could I do it wrong!” “…I used to race. I know. Whatever I feel comfortable with, you know, or whatever I feel comfortable with would be the same for this one (child)… so far it hasn’t done me wrong.”
	*The “good-enough” Parent:* May or may not have experience and demonstrates moderate to little confidence in perceived installation knowledge/ability/skill but resides in belief that the child will be fine and his/her efforts are good enough to “safely” transport child from one point to another. May or may not exhibit assistance seeking behaviors to improve car seat instillation over time but will utilize information obtained/received.	“You put the seat belt underneath or you put the seat belt around and that’s that.” “I would have just tried (installing car seat) the best way I could, use the manual and install it that way. I wouldn’t have used the video.”
	*Secondary caretakers:* actor that at times installs or assists the primary car seat installer (above caretaker roles) in the installation process. (This may include a spouse, grandparent/parent, family, friends, babysitters, etc.)	“The problem for me is when he (father) has to go to work and we have only one car and we need to ask for a ride and they have to install the car seat.” My in-laws put the kids in the car since I was meeting them somewhere and my son’s thing was literally down to his stomach. I was, “are you kidding me?” but they don’t know.
	*Non-compliant, car seat-resistant child:* Child actor that resists or experiences disease being in a car seat. Parent/Caretaker often has to decide whether the parent will be permissible or obstinate in regards to safety instructions/rules.	“He just doesn’t like the rear facing. So I know doctor recommends 2 years for the rear facing but I had to change it because I can’t bear…” Child says—“Mommy why are you doing that? It’s choking me. It hurts my neck. I don’t like it.” Always saying, “mommy, are you sure? Mommy come and take it off. Sometimes I look and the arm is out…
	*Compliant child*: Actor that facilitates easy car seat wearing.	“My son…completely loves being in his car seat…he loves car rides. He’s like this the whole time. His legs are just going and he’s making so much noise and then 20 min later he’s out like a light. Every single time. Every time.”
*Child car seat development timeline:* The sequential order of child growth and corresponding car seat over a period of time the duties, attitudes, challenges, and behaviors exhibited by the actors as the child progresses from one car seat to another.	*Prenatal period:* The period in which the parent is inundated with new information for prenatal and infancy care and has the task of selecting and purchasing the child’s first car seat as well as making initial contact with new information to assist with the instillation.	“… I was learning about it, in the hospital before the baby gets here. Do you have a car seat? If the information was there beforehand, you could take your own time and learn about then. If I had it, ahead of time and had more information…”
	*New-born/immediate post-birth period*: Short period in which the parent completes the instillation process for the first time.	“I remember me sitting in the back seat with the baby hold in on to the car seat.” Just reading the instructions is very important or just knowing how it is that important…the day leaving the hospital I had no idea. If it weren’t for that nurse standing right there I probably would have went up to the neonatal intensive care unit and like “Does anybody know what to do?”
	*Infancy period-rare facing:* the period in which the parent utilizes method(s) of instillation leading up to the need for new car seat. In this period parents may be provided instillation information by community agents, and/or exhibit instillation assistance seeking behaviors and utilizes various methods to improve car-seat fit.	“The baby ones are really complicated cause after you put them in then you get these little two straps here some little strap here, a little harness here…”
	*Transition:* the period in which the child begins to outgrow the rare-facing car seat and parent encounters new information for car seat switch. The parent must determine the next car seat to be used.	“Well when I switched him from the actual newborn car seat I asked when I was at an appointment because it came a time when he was too long. I’m like should he still be in this little car seat. He was just too long his legs were like…so.” “And then we have another car seat for another car and that ones completely different of course.”
	*Toddler-forward facing:* the child is growing older and parent is getting used to new car seat behavior pattern and attitudes.	“Those seats (front facing seat) made me now miss the base because it was so easy to drop it in and didn’t matter if you had two cars the base in each car and we just clicked it back and forth. Now we don’t have like that…we don’t have four car seats two for each car we just basically have two for mine and then like we switch it to my husbands and the big ones are big there heavy there bulky…” Parent with child in convertible: “They’re big, they’re heavy, and I don’t have strong enough hands to do it… then there’s that top clip which I just don’t enjoy which is another hand strength issue.”
*Child-car seat instillation continuum:* the attitudes, behaviors and events exhibited by caretaker(s) as the child progresses across the child-car seat development timeline.	*Preparation:* the parent receives/obtains new information to assist with instillation and is tasked to select and purchasing car seat.	“Yeah I think a lot of my girlfriends. I mean I was like 30 when I had my first daughter. All my friends been having kids for a long time… I got some information from them and looked on the Internet. I mean I read a lot when I was pregnant. I think having it available as early as possible.”
	*Execution:* parent utilizes instillation knowledge and completes first-time instillation process. This may be done with the aid of community agents, and electronic resources.	“When we came home from the hospital, her dad was trying to put…we had the two in one. Like it was the baby car seat, and then it will turn into an infant seat, but some reason it was so difficult. Like was so big to be for an infant. After a while, we didn’t use it… I went and got the regular just infant one, we used that one, and after the infant one we used…I switched over to the taller one because she got too big for the infant one.” “I couldn’t get it to be level…I didn’t know how to loosen it so that the chair wasn’t kind of back up against like the seat. So it was a disaster.”
	*Evaluation*: parent assesses effectiveness and efficiency of initial installation and either moves to resolution stage or seeks new methods of instillation.	“We came home from the hospital and it was in my dad’s car and to put it in my apartment…”I don’t know about this “guys we’re gonna have them check this.” “I actually went to a fire/police station…a cop was there he installed it…he made us do it in front of him the right way so that’s how I got my experience with her seat.”
	*Resolution:* parent accepts the instillation method of choice.	“I was trying to put it on the wrong way. Yeah, it was bad but it got better…”
*Instillation facilitators:* the various technological and community agents/resources that provide the caregiver(s) instillation information and assist in successfully installing car seats across the child-car seat continuum.	*Community agents/resources:* that provide the caregiver(s) instillation information and assist in successfully installing car seats across the child-car seat continuum.	Family and Friends: “I was actually at a friend’s house and she was helping me put stuff in my car and she noticed that both of my seats were forward facing and she knew that my two year old was less than two at the time. She said, “why is he in a forward facing seat?” and I said, “Because his legs are too long he couldn’t sit” and then she said, “did you know that his spine’s not completely developed and that’s why?” Strangers: “One time I took her to the WIC office when she was a baby… The base was shifting… I took the car seat out with the base too and there was a lady who also went to the WIC office and I have her in the stroller and she said, “oh you don’t have to take this whole thing. You leave that in the car.” Then she was showing me how… so sometimes you get information from friends, strangers” Hospital Staff: The ladies up in the NICU unit advised and they went by standards and gave me a list of recommendations. Hands down had a checklist of like safety mechanisms how easy it is to install or anything like that. Safety first had all the checks. Pediatricians/pediatrician office staff: “I’m guided most of the time by my son’s pediatrician.” “We go to our pediatrician for everything so definitely as backup having the pediatrician.” Safety checkpoints: “I actually went to a fire/police station… a cop was there he installed it… he made us do it in front of him the right way so that’s how I got my experience with her seat.”
	*Technological resources:* videos, websites, etc. that provide the caregiver(s) instillation information and assist in successfully installing car seats across the child-car seat continuum.	Manufacture resources (CD/DVD/website): “My car seat came with a video or a CD, I watched that, and I got the fire department to check it.” “I did look at the ones that came from the manufactures and then I looked at other people just regular people that post on YouTube.” Non-manufacture Videos/tutorials: “You tube shows you how to everything so if you can x-out YouTube and just go right into the app for videos, that will be perfect.” The hospital video about car seat safety—“it showed the importance of installing car seats right and what could happen… I was like I have to do everything right cause (the video) completely scared me.” Online forums: “I heard of (Safe Kids) from the Internet from like a forum group. Online reviews: “I was transitioning to a bigger car. I did move online to figure out which ones have the best reviews.” “Which car seat had the best reviews. Mostly from Wal-Mart and Target. That’s where I was deciding to go” Online search engines American pediatric—“He (the father) is a fan of the American pediatric thing so he went online. He went to credible… that’s how we got the brand we got.” You can Google anything now. Can’t lose. The websites can tell me some things I don’t know. It’s out there.
*Instillation barriers:* The circumstances or obstacles that prevents the caregivers from successfully purchasing and installing the car seat in the vehicle.	*Lack of information*: caretakers noted lack of knowledge about instillation due to difficulty with manufacture instructions as well as lack of knowledge about child development, transition guidelines, and child-seat safety laws:	Lack of information of which seat to purchase “When you go to buy the seat… sometimes you go to the store and there’s all these seats and no info and you don’t know what you’re looking at.” Trouble reading instructions: “I get to the first paragraph. The pictures are great, I love pictures, and then it goes haywire after the first paragraph.” Lack of knowledge about transitioning seats between cars “I was confident in the hospital staff, you know, checking before we leaved but once it was my turn to transition to another car then I wasn’t confident.” Lack of knowledge about transitioning between seats (infant and toddler seats) “I was confident in the hospital staff, you know, checking before we leaved but once it was my turn to transition to another car then I wasn’t confident.“ Lack of knowledge of laws and guidelines “My kid’s legs are too long now. I’m gonna turn em around but his spine’s still not developed… I feel like there’s need to be educated as to like—okay you’re telling us these rules, tell us why? What in their development?” Lack of knowledge of resources for information and instillation assistance “I’ve never even known that the Fire Company or police stations… could help with (instillation).”
	Adjusting/maneuvering car seat: emphasis was noted among care takers regarding the difficulty adjusting and maneuvering straps, latches, etc. to ensure secure instillation.	Straps and latch “His new one that he has in there now has 4 or 5 safety straps they go they attach to underneath the seat to the safety mechanism in your seat and then the bars. You literally have to take apart my whole back seat just to get his car seat out” “My hands aren’t strong enough… we use the latch system and which is nice but I don’t have strong hands. Like, “I can’t find it” and eventually I get it in there. Unsteady base “I couldn’t get it to be level…I didn’t know how to loosen it so that the chair wasn’t kind of back up against like the seat. SO it was a disaster”
*Proponents of App Use:* factors that motivate caretakers to use the car seat app.	*Easily accessible information.*	“Apps are so much easier I think…you don’t have to remember the website” “…You’re outside, so I have my phone with me. I wouldn’t have my computer outside with me. It would just be right there and I could check it and would be good to go…”
	*Predictability*	“I like the app. like I know what to expect”
*Deterrents of app use:* factors that deter students from using the car seat app.	*Some caretakers voiced not being technologically savvy.*	I actually don’t use apps. I have a smartphone but I don’t use apps … I’m also old school I like to discuss more phone like last year. I actually have flip phone…
	*Occasional use.*	“I’m not going to use it like for every day. Just like one or two times and that would be it” “It would be good for a first child but after 3 or 6 months…yeah…unless you could show or have some kind of insight on different brands because some brands are different. Some have different types of latches… The hardest part for me is when you pull it tight. Those things are different.”
	*Belief the web is more user-friendly and will provide more information.*	“I don’t use the mobile…I go to safari because I think it has more information and it’s easier for me to navigate than the mobile friendly version.”
Care-giver app preferences: participant identified preferences that would motivate use of car seat app.	*Factoids: facts about car seat instillation as well as car seat safety rules, law, guidelines.*	“Things that are important, things that will save my child from death in an accident and they could be safe.” “Like let them know the dos and don’ts—like the coat and things like that.”
	*Car seat-vehicle compatibility.*	“Like what type of car seat for like a mini-van vs. a sedan or what fits better. Cause we have 2 I guess they’re bucket seats and there’s one seat in the back and the car seat sit differently in the back than they do in the bucket.” “I think they will be really useful cause like the fact that you found the best type of car seat for my car. An app that told you that and them you go look at it and it can give you like see how to install it or I hate to make ads for people but even tell you where you can get them.”
	*Video tutorials: tutorial of installing various seats in various types of cars as well how to adjust straps and position child in seat.*	“I think one for each stage…the seat with the booster, the seats with the base that you carry around, and then each stage up and so you can just click on my child is using this seat this is how to install this one or you know. Plus that would help with knowing whether or not your child is in the right seat cause if they show a video and you are putting your 1.5 years old in a seat that a four year old is in in the video then you’d be like oh maybe my child shouldn’t be in this seat after all.” “Actually installing it…like pulling out the strap and saying look for these particular things and showing up pictures of what we’re looking for and how to push them to connect… The simplest thing is sometimes not as simple to some people like maybe. I think just clamping it is very simple but to someone else it’s not and vice versa, you know.”
	*Access to online forums or parent created car seat reviews.*	“Yeah and I think it would be good if people could write personal reviews cause I would defiantly like to hear what people have to say.”

**Table A2:** Parents’ use of child restraints.

Selected Items from Survey	N (%)
*How did you first learn how to install a car seat?*	
Manual that came with the car seat	683 (54.6)
Car manual	30 (2.4)
Certified safety technician	26 (2.1)
Car seat “check clinic”	83 (6.6)
Nurse/Care provider at the hospital	73 (5.8)
Friend	51 (4.1)
Family Member	235 (18.8)
Brochure	8 (0.6)
Internet	41 (3.3)
Other	20 (1.6)
*In general, who primarily installs your child's car seat?*	
Self	879 (70.3)
Co-parent	233 (18.6)
Other	139 (11.1)
*About how often do you install a car seat?*	
At least once a day	155 (12.4)
At least once a week	315 (25.2)
At least a month	137 (11.0)
I leave the car seat installed in my car	644 (51.5)
*It is a hassle to install a car seat.*	
Strongly Agree	107 (8.6)
Agree	345 (27.6)
Neither Agree nor Disagree	274 (21.9)
Disagree	323 (25.8)
Strongly Disagree	202 (16.1)
*I have my child ride in a car seat.*	
Always	1198 (95.8)
Sometimes	46 (3.7)
Never	7 (0.6)
*I ask other people to have my child use a car seat when they are driving.*	
Always	1144 (91.4)
Sometimes	85 (6.8)
Never	22 (1.8)
*I have my child ride in a car seat even when I drive his/her friends who do not have car seats.*	
Always	1123 (89.8)
Sometimes	76 (6.1)
Never	52 (4.2)
*I have someone check to see if my car seat is installed the right way.*	
Always	380 (30.4)
Sometimes	519 (41.5)
Never	352 (28.1)
*How often are you able to get your child buckled into the car seat, even if he/she resists?*	
Always	1148 (91.8)
Most of the time	83 (6.6)
Half of the time	15 (1.2)
Rarely	3 (0.2)
Never	2 (0.2)

**Table A3:** Parents’ car seat installation resources.

Selected Items from Survey	N (%)
*Where do you usually get information (such as news, facts, or tips) about car seat safety?*	
Primary care provider (like your child‘s doctor or your normal doctor)	425 (34.0)
Friends	474 (37.9)
Family member	519 (41.5)
Book/Brochure	355 (28.4)
Class	49 (3.9)
Internet	920 (73.5)
Other	53 (4.2)
*How easy is it for you to find information (such as news, facts or tips) about car seats?*	
Easy	1028 (82.2)
Neither easy nor hard	211 (16.9)
Hard	12 (1.0)
*When you search for information about car seats, how satisfied are you with the information you find?*	
Satisfied	759 (60.7)
Somewhat Satisfied	405 (32.4)
Neutral	77 (6.2)
Somewhat Dissatisfied	9 (0.7)
Dissatisfied	1 (0.1)

**Table A4:** Parents’ use and preferences around digital media and devices.

Selected Items from Survey	N (%)
*Do you own a mobile phone?*	
Yes	1210 (96.7)
No	41 (3.3)
*Which of these devices do you use most often to get onto the Internet?*	
Mobile phone	347 (27.7)
Tablet	108 (8.6)
Computer (desktop or laptop)	796 (63.6)
*Have you ever used your mobile device to do any of the following?*	
Send or receive email	1066 (85.2)
Send or receive text messages (SMS)	1136 (90.8)
Take a photograph	1128 (90.2)
Use the Internet	1097 (87.7)
Look for health or medical information online	777 (62.1)
Download a software application or “app”	1002 (80.1)
I don’t use a mobile device	35 (2.8)
None of the above	15 (1.2)
*Would you ever consider downloading an app on your smartphone or tablet device to help manage or look for information about your child's health and safety?*	
Yes	849 (67.9)
No	402 (32.1)
*If there were a mobile app for your smartphone or tablet device that included information about car seat installation and car seat safety, would you choose to use it?*	
Yes	453 (36.2)
No	251 (20.1)
Maybe	547 (43.7)
*A mobile app about car seats would be simpler to use than the car seat manual.*	
Strongly Agree	223 (17.8)
Agree	379 (30.3)
Neither Agree nor Disagree	330 (26.4)
Disagree	232 (18.5)
Strongly Disagree	87 (7.0)
*A mobile app about car seats would be more convenient to use than the car seat manual.*	
Strongly Agree	251 (20.1)
Agree	447 (35.7)
Neither Agree nor Disagree	270 (21.6)
Disagree	188 (15.0)
Strongly Disagree	95 (7.6)
*A mobile app about car seats would be easier to access than a website.*	
Strongly Agree	237 (18.9)
Agree	421 (33.7)
Neither Agree nor Disagree	336 (26.9)
Disagree	185 (14.8)
Strongly Disagree	72 (5.8)
*A mobile app about car seats would not be useful after the first few uses.*	
Strongly Agree	244 (19.5)
Agree	366 (29.3)
Neither Agree nor Disagree	286 (22.9)
Disagree	232 (18.5)
Strongly Disagree	123 (9.8)
*Using a mobile app for car seat installation would be useful to me.*	
Strongly Agree	176 (14.1)
Agree	410 (32.8)
Neither Agree nor Disagree	352 (28.1)
Disagree	221 (17.7)
Strongly Disagree	92 (7.4)
*I would prefer to use a website over a mobile when looking for information (such as news, facts, or tips) about car seats.*	
Strongly Agree	287 (22.9)
Agree	389 (31.1)
Neither Agree nor Disagree	357 (28.5)
Disagree	179 (14.3)
Strongly Disagree	39 (3.1)
